# Inspiration for COVID-19 Treatment: Network Analysis and Experimental Validation of Baicalin for Cytokine Storm

**DOI:** 10.3389/fphar.2022.853496

**Published:** 2022-03-08

**Authors:** Jia You, Huawei Li, Peng Fan, Xi Yang, Yuanfeng Wei, Lingnan Zheng, Zhaojun Li, Cheng Yi

**Affiliations:** ^1^ Department of Oncology, Hospital of Chengdu University of Traditional Chinese Medicine, Chengdu University of Traditional Chinese Medicine, Chengdu, China; ^2^ Department of Respiratory and Critical Care Medicine, Chongqing Traditional Chinese Medicine Hospital, Chongqing, China; ^3^ Department of Medical Oncology, Cancer Center, West China Hospital, Sichuan University, Chengdu, China; ^4^ Department of Radiation Oncology, Hainan Affiliated Hospital of Hainan Medical University, Haikou, China

**Keywords:** baicalin, cytokine storm, COVID-19, network analysis, molecular docking

## Abstract

**Background:** Cytokine storm (CS) is a systemic inflammatory syndrome and a major cause of multi-organ failure and even death in COVID-19 patients. With the increasing number of COVID-19 patients, there is an urgent need to develop effective therapeutic strategies for CS. Baicalin is an anti-inflammatory and antiviral traditional Chinese medicine. In the present study, we aimed to evaluate the therapeutic mechanism of baicalin against CS through network analysis and experimental validation, and to detect key targets of CS that may bind closely to baicalin through molecular docking.

**Method:** Access to potential targets of baicalin and CS in public databases. We constructed the protein-protein interaction (PPI) network of baicalin and CS by Cytoscape 9.0 software and performed network topology analysis of the potential targets. Then, the hub target was identified by molecular docking technique and validated in the CS model. Finally, GO and KEGG pathway functional enrichment analysis of common targets were confirmed using R language, and the location of overlapping targets in key pathways was queried via KEGG Mapper.

**Result:** A total of 86 overlapping targets of baicalin and CS were identified, among which MAPK14, IL2, FGF2, CASP3, PTGS2, PIK3CA, EGFR, and TNF were the core targets. Moreover, it was found that baicalin bound most closely to TNF through molecular docking, and demonstrated that baicalin can effectively inhibit the elevation of TNF-α *in vitro* and *in vivo*. Furthermore, bioenrichment analysis revealed that the TNF signaling pathway and IL-17 signaling pathway may be potential key pathways for baicalin to treat CS.

**Conclusion:** Based on this study, baicalin was identified as a potential drug for the alleviation of CS, and the possible key targets and pathways of baicalin for the treatment of CS were elucidated to reveal the main pharmacological mechanisms.

## Introduction

Cytokine storm (CS) is a systemic inflammatory syndrome with an increased level of circulating cytokines and hyperactivation of immune cells caused by pathogens, autoimmune diseases, and carcinomas, leading to multi-organ failure, and even life-threatening conditions (Fajgenbaum and June 2020; Kim et al., 2021). Whereas the initial drivers may vary, the pathological mechanisms and clinical manifestations of CS often converge and overlap (Fajgenbaum and June 2020). Among them, pathogen-induced CS has the large ripple effect, and seriously endanger public health. Certain highly pathogenic infectious coronaviruses, which were known as Severe Acute Respiratory Syndrome Coronavirus (SARS-CoV) and Middle East Respiratory Syndrome Coronavirus (MERS-CoV), evoked prolonged and excessive cytokine and chemokine responses, followed by CS, with consequent imbalance of immune damage repair processes and high mortality (Oldstone and Rosen, 2014; Channappanavar and Perlman, 2017). Similarly, high-level cytokines (*e.g.*, IL-1β, IL-6, IL-8, IL-10, IFN-γ, and TNF-α, etc.) were registered in the blood of Coronavirus Disease 2019 (COVID-19) patients (Rothan and Byrareddy, 2020; Ruan et al., 2020). COVID-19-associated CS provoked strong inflammatory immune responses. Meanwhile, an increasing number of studies have observed that CS was the critical driver of acute respiratory distress syndrome (ARDS) and multi-organ failure, even death, in COVID-19 patients (Li et al., 2020; Mehta et al., 2020). In parallel, novel severe acute respiratory syndrome coronavirus 2 (SARS-CoV-2) variants (*e.g.*, Delta and Omicron) were still emerging and rampant in countries around the world (Callaway and Ledford, 2021; Dougherty et al., 2021). Hence, modulating and alleviating the CS can be a critical strategy to reduce COVID-19-related comorbidity or mortality. Presently, CS has been primarily managed with cytokine inhibitors (*e.g.*, Tocilizumab, Etanercept, and Ruxolitinib) and glucocorticoids (*e.g.*, dexamethasone) to arrest the hyperactivated immune response (Molinaro et al., 2020; Kim et al., 2021). However, it also blocked the removal of the virus from the body and increased the risk of repeat infection (Nile et al., 2020; Kim et al., 2021). Therefore, new therapies continue to be in extremely high demand for the treatment of COVID-19 related CS.

Baicalin (7-D-Glucuronic Acid-5,6-Dihydroxyflavone) is a biological active flavonoid of natural origin obtained primarily from the roots of *Scutellaria baicalensis Georgi*, an East Asian skullcap plant, which contains 10.11% baicalin and is widely used for the clinical treatment of dysentery, respiratory infections, and inflammatory diseases in traditional Chinese medicine (TCM) (Zhao et al., 2016; Dinda et al., 2017). It has been demonstrated that baicalin has anti-SARS virus activity by plaque reduction assays (Chen et al., 2004). In addition, baicalin can directly inhibit SARS-CoV-2 RNA-dependent RNA polymerase (RdRp) activity and exhibit significant antiviral activity against SARS-CoV-2 *in vitro* (Zandi et al., 2021). Moreover, it was reported that baicalin can suppress angiotensin-converting enzyme 2 (ACE2) activity to inhibit the attachment of SARS-CoV spike protein (S protein) to ACE2, which was the entrance of SARS-CoV-2 into host cells via S protein, reducing infections (Yang et al., 2020; Boozari and Hosseinzadeh, 2021). In influenza A-infected A549 and MDCK cells, baicalin also could increase IFN levels, resulting in a reduction of pro-inflammatory cytokines production (Nayak et al., 2014). Meanwhile, baicalin greatly attenuated inflammatory cell infiltration and decreased the levels of pro-inflammatory cytokines tumor necrosis factor α (TNF-α), IL-6, and IL-1β by regulating a variety of molecular mechanisms including Nuclear erythroid factor 2 (Nrf2)/heme oxygenase 1 (HO-1), CX3CL1-CX3CR1 axis and TLR4/MAPKs/NF-κB, to defend against lipopolysaccharide-induced severe lung injury (Ding et al., 2016; Meng et al., 2019; Long et al., 2020). Baicalin also boosted the differentiation and modulatory activity of Foxp3^+^ Treg cells, promoted TGF-β secretion, and significantly inhibited the production of inflammatory cytokines (Yang et al., 2019). However, there is not yet a complete elucidation of whether baicalin can provide a basis for COVID-19 treatment by modulating immune status and preventing CS.

**FIGURE 1:**
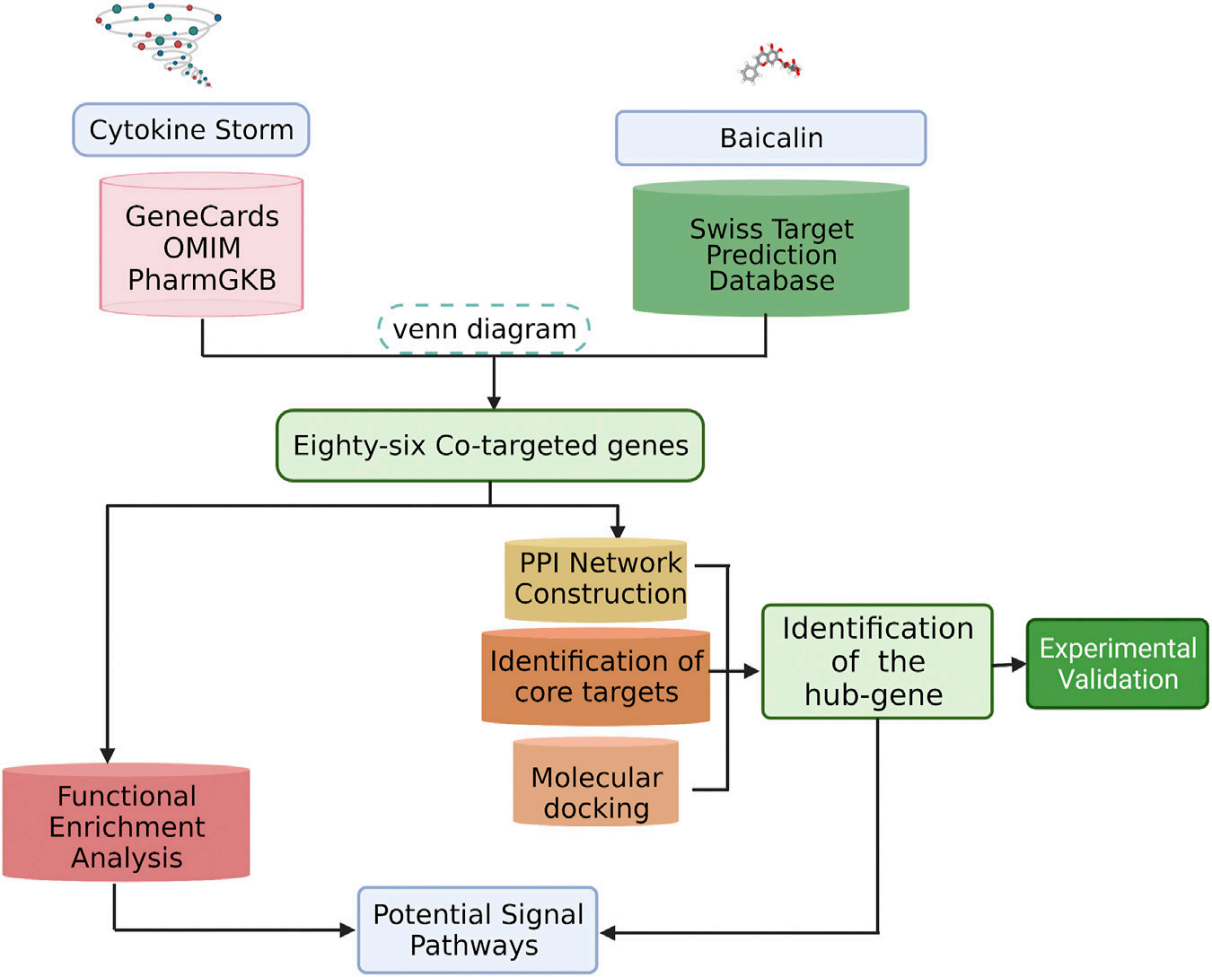
Flowchart of this study. We identified potential biological targets for baicalin and characterized its core biological targets against CS. A PPI map of baicalin against CS was generated. Our analysis revealed the pharmacological effects and molecular pathways of baicalin against CS.

Here, we would predict the potential targets and signaling pathways of baicalin for the treatment of CS, and analyze the relationship between active ingredients and targets with bioinformatics and network analysis. Subsequently, biological validation was conducted by molecular docking techniques and experimental models. The results are expected to provide further insight into the mechanism of baicalin in inhibiting CS, and provide a reference for the potential molecular mechanism of hyperimmune regulation. The overall workflow of this study was presented in Figure 1.

## Materials and Methods

### Screening Corresponding Targets of Baicalin and CS

As shown in Figure 1, we downloaded the two-dimensional (2D) molecular structure, PubChem CID and SMILES structural formula of baicalin from PubChem (https://pubchem.ncbi.nlm.nih.gov/), the world’s greatest free chemical information database (Kim, 2016). To forecast the potential targets of baicalin, we collected the relevant target proteins in the Swiss Target Prediction Database (http://swisstargetprediction.ch/) by the SMILES structural formula (Gfeller et al., 2014). Simultaneously, target genes associated with CS were obtained from GeneCards (Rebhan et al., 1997) (https://www.genecards.org/), OMIM (Amberger and Hamosh, 2017) (https://omim.org/), and PharmGKB (Hewett et al., 2002) (https://www.pharmgkb.org/) databases according to keywords such as “cytokine storm and cytokine release syndrome”. After that, the overlapping target genes were identified by analyzing the two clusters of CS and baicalin and mapping the Venn diagram with Venny 2.1, a free online data analysis platform (https://bioinfogp.cnb.csic.es/tools/venny/index.html).

### PPI Network Construction of the Mutual Target

The protein interaction relationships were acquired by importing the mutual target genes of CS and baicalin into the String database (https://www.string-db.org/) with the species restriction of “*Homo sapiens*” and setting the confidence level >0.4 (von Mering et al., 2003). And, the results were built the PPI networks which were visualized by Cytoscape9.0. Then, the core targets in the PPI network that qualified for the screening criteria (Li et al., 2014) were accessed by CytoNCA(http://apps.cytoscape.org/apps/cytonca), a network topology analysis plug-in (Tang et al., 2015). In parallel, the number of contiguous nodes for each gene was computed by R language software (V3.6.1), and the significance of the genes was assessed based on the number of contiguous nodes.

### Molecular Docking

Molecular docking was commonly applied to assess the possibility of molecular structural interactions and forecast the ligand-protein binding mode based on the 3D structure (Zígolo et al., 2021). As a further insight into the strength and mode of interaction between baicalin and the core gene, we prepared chemical structures of baicalin in PubChem database (https://pubchem.ncbi.nlm.nih.gov/compound/10114) as well as received protease crystal structures of the target gene from RCSB Protein Data Bank (PDB, http://www.rcsb.org/), and ChemBio3D Ultra 14.0 was used to minimize the energy of baicalin’s structure to obtain the most stable molecular conformation. The SURFLEX-Dock mode from SYBYL-X2.0 software was utilized for molecular docking and scoring (Jain, 2003; Ivanov et al., 2006). SURFLEX-Dock was a high accuracy and fast docking technology that represented an advancement in flexible molecular docking (Jain, 2003; Attique et al., 2019). In the SURFLEX-Dock, the docking results were expressed as scoring functions. The total score function integrated polarity, hydrophobicity, entropy and solubility, and directly reflected the strength of the interaction between the drug molecule and the receptor (Rarey et al., 1996). Higher values were associated with more stable docking complexes, indicating that the drug molecule was better matched, and bound to the receptor. The C Score (Consensus Score) was a consistent scoring function for ligand-receptor affinity, including D Score, PMF Score (Potential of Mean Force Score), G Score, and CHEM Score functions, etc. The C Score scores ranged from 1 to 5, with the best C Score being 5. The G score was based on hydrogen bonding and intramolecular forces. The D score, Chem score and PMF score reflected the van der Waals force and electric field interaction, the hydrogen bonding energy and the Helmholtz free energy between the drug molecule and the receptor, respectively (Clark et al., 2002).

### Anti-CS of Baicalin *in vitro*

MH-S cell lines (CRL-2019, ATCC) were suspended in RPMI-1640 medium with 10% fetal bovine serum and antibiotics (100 units/ml penicillin and 100 μg/ml streptomycin). The cells were seeded in 96-well plates at a density of 1 × 10^4^/ml and incubated with different concentrations (12.5, 25, 50, and 100 μg/ml) of baicalin (CAS: 21967-41-9, HPLC≥95%, meilunbio) for 24 h at 37°C and 5% CO_2_. Then, the cell viability was calculated by CCK-8 kit assay (MA0218, meilunbio). The specific procedure followed the instructions of the CCK-8 kit. Subsequently, MH-S cells were cultured in 48-well plates at a density of 5 × 10^4^ cells for 24 h, and then co-incubated with 10 μg/ml baicalin for 24 h. 50 ng/ml dexamethasone (Dex, H12020514, and Tianjin Jinyao Pharmaceutical Co.) was used as a positive control. After the addition of 100 ng/ml lipopolysaccharide (LPS, L2880, Sigma-Aldrich China, Inc.) for 4h, the cell supernatant was collected and analyzed the concentration of TNF-α by enzyme-linked immunosorbent assay (ELISA). The specific procedure followed the instructions of the TNF-α ELISA kit (1217202, Dakowei Biotechnology Ltd.).

### Anti-CS Baicalin *in vivo*

Male C57BL/6J mice (8 weeks old, 23 ± 2 g) were purchased from Beijing HuafuKang Biotechnology Co., Ltd. and kept in an SPF-grade animal room at the West China Hospital Animal Experiment Center, Sichuan University, where the temperature was approximately 22 ± 1°C and the relative humidity was 55–60%, with alternating light every 12 h to simulate day and night. The mice were free to drink water and eat. After 1 week of aclimatization, the mice were randomly divided into control group, LPS group, Dex (1 mg/kg) group and baicalin (200 mg/kg) group. After 5 days of baicalin gavage and 3 days of Dex intraperitoneal injection, each group was given 5 mg/kg LPS intratracheal drip to trigger CS except for the control group, which was given the same saline intratracheal drip. Four hours later, bronchoalveolar lavage fluid (BALF) was taken to detect the concentration of TNF-α by ELISA. The intratracheal drip and BALF collection steps were performed as described in the previous study (Chen et al., 2017).

### GO Function Enrichment and KEGG Pathway Enrichment Analysis

The shared target gene symbols of baicalin and CS obtained from the screening were converted into gene IDs, which were subjected to gene ontology (GO) functional analysis and Kyoto Protocol Encyclopedia of Genes and Genomes (KEGG) pathway enrichment analysis by the R language, with *p* < 0.05 representing statistically significant differences. Annotation of GO terms consisted of biological process (BP), cellular component (CC), and molecular function (MF) categories. The KEGG-pathway database (https://www.kegg.jp/kegg/) for pathway mapping was applied.

## Result

### Potential Baicalin-Related Target Genes in CS

**FIGURE 2:**
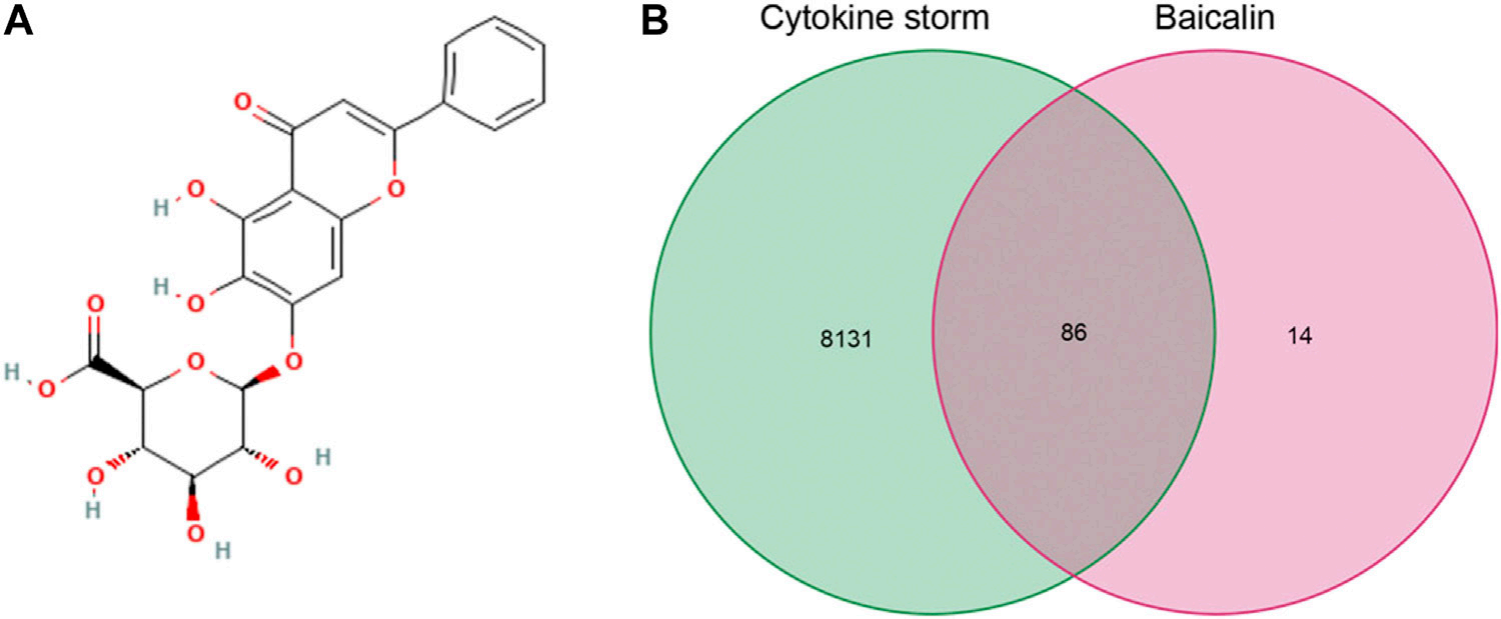
Baicalin and CS information acquisition. **(A)** 2D molecule structure of baicalin; **(B)** Venn diagram of baicalin and CS targeted genes.

We downloaded the 2D molecular structure and SMILES {C1 = CC = C(C=C1)C2 = CC(=O)C3 = C(C(=C(C=C3O2)OC4C(C(C(C(O4)C (=O)O)O)O)O)O} of baicalin from PubChem (Figure 2A), the PubChem CID of baicalin was 64982. The chemical formula and molecular weight of baicalin are C_21_H_18_O_11_ and 446.4 g/mol, respectively. Then, one hundred major target genes on baicalin were accessed from the Swiss target prediction database. Meanwhile, a total of 8217 CS-related target genes were also collected from GeneCards, OMIM and PharmGKB databases. Consequently, a total of 86 overlapping target genes (Figure 2B, Supplementary File S1) were identified from the two clusters of target genes for subsequent analysis of the potential significance of baicalin on treatment of CS.

### Network Analysis of Potential Therapeutic Targets

**FIGURE 3:**
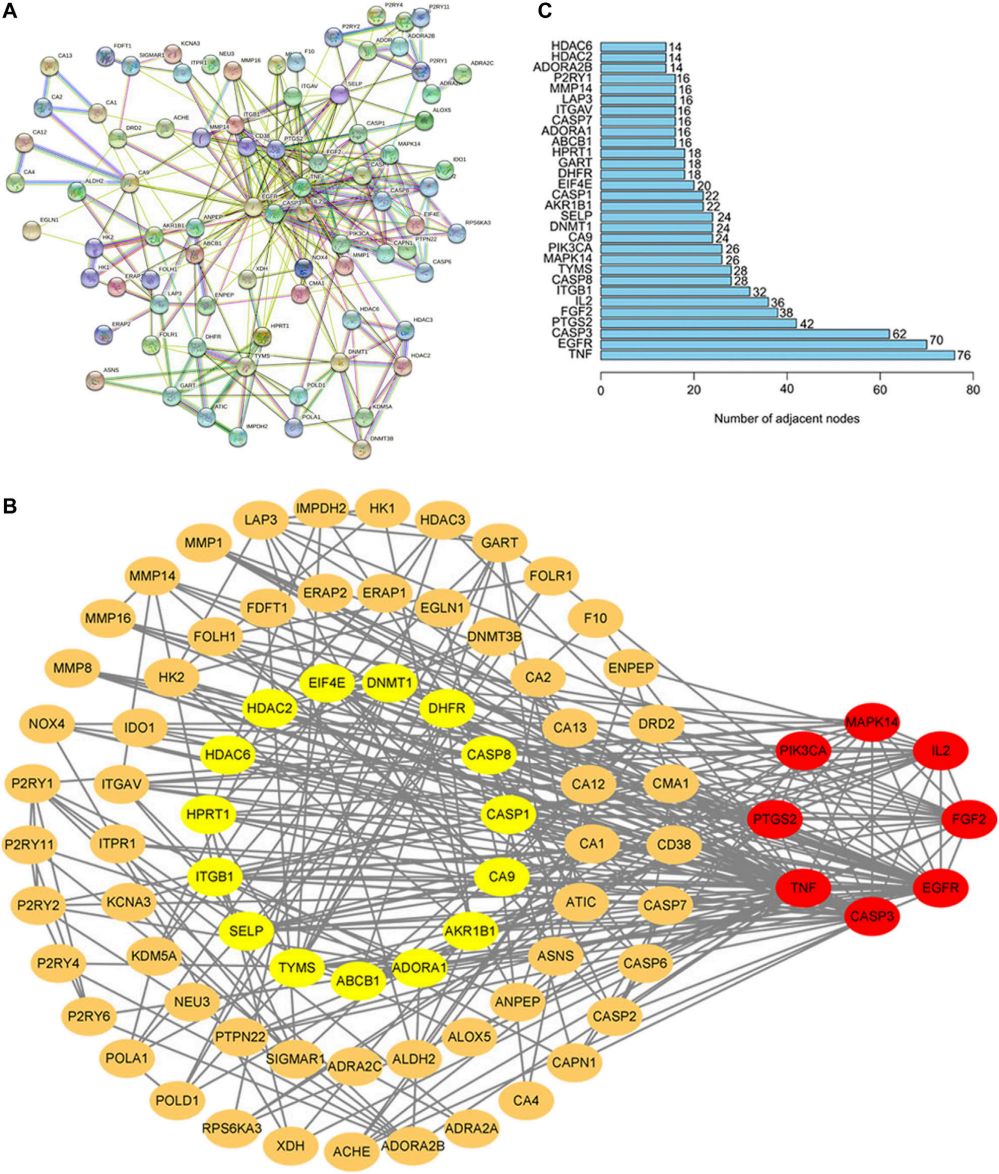
PPI Network Analysis in the Common Target of Baicalin and CS. **(A)** In STRING, the nodes stand for proteins and the junctions represent the interactions between proteins. The more junctions there are, the greater the degree of connection is. **(B)** The core genes screened by Cytoscape 9.0. The node color was regulated by the degree centrality, and the red node had a higher centrality; **(C)** In the core gene bar diagram, the horizontal coordinates indicate the number of genes and the vertical coordinates indicate the gene names.

PPI network of potential target genes for baicalin to treat CS was presented in Figure 3A. Based on the topological characteristics of the network nodes, MAPK14, IL2, FGF2, CASP3, PTGS2, PIK3CA, EGFR, and TNF were identified as the important nodes in the network (Figure 3B). Furthermore, it was confirmed that the number of genes in TNF and EGFR target nodes (76 and 70, respectively) was significantly higher than that in other nodes based on the information of the number of genes by R language (Figure 3C). It was the number of adjacent nodes that represented the relationship of genes in the network. The higher the number of gene connections were, the more important the relationship of the gene was in the network, indicating that the gene was a potential key target gene for the treatment of CS. Therefore, TNF and EGFR were more important in the protein interaction network, and they would be the critical target genes for the therapeutic effect of baicalin and its association with CS.

### Molecular Docking Analysis of Baicalin With Core Targets

**FIGURE 4:**
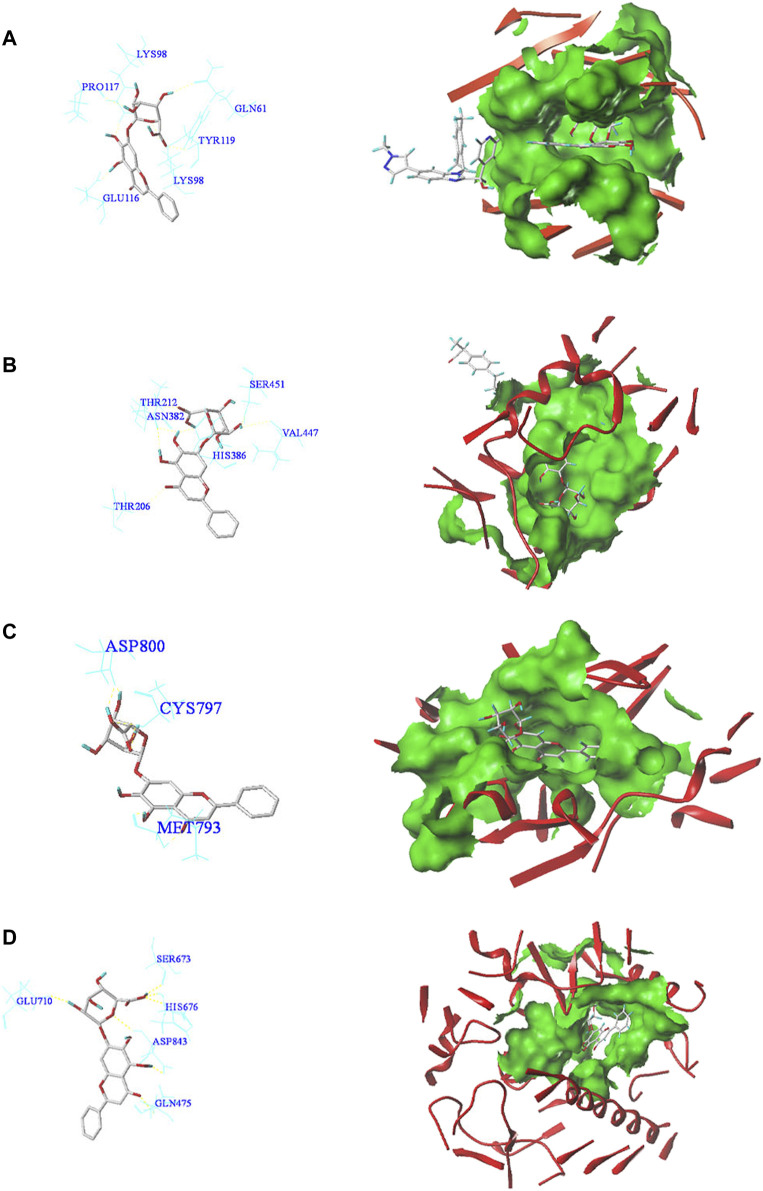
Molecular docking patterns of core target receptors with baicalin in two dimensions and three dimensions. **(A)** TNF protein-baicalin; **(B)** PTGS2 protein-baicalin; **(C)** EGFR protein-baicalin; **(D)** PIK3CA protein-baicalin.

In order to reveal the interaction between baicalin and CS-related core targets, we further analyzed and evaluated the binding affinity of baicalin and core targets by molecular docking with SYBYL-X2.0 software. It was found that the docking total scores of baicalin with MAPK14, IL2, FGF2, CASP3, PTGS2, PIK3CA, EGFR, and TNF were in the range of 2.44–9.24 (Table 1). In general, as the total score is higher, the more stable is the binding between the ligand and the target protein receptor. When the total score is greater than 5, the molecule has good binding activity to the target (Figure 4). In addition, Consensus Score (C Score) is mainly utilized to rank the affinity of the ligand bound to the active site of the receptor, with higher scores indicating stronger binding affinity. Baicalin had the highest docking total score (9.24) and C Score (4.0) with TNF, indicating that baicalin was seamlessly situated within the binding site with TNF. TNF may be a potential therapeutic target of baicalin.

**TABLE 1:** The docking score and C Score of baicalin and key target proteins.

Target	PDB ID	Total score	D score	PMF score	G score	CHEM score	C score	Amino acid residues
TNF	6OP0	9.24	-160.82	-100.62	-236.93	-22.96	4	LYS98 GLN61 TYR119 PRO117 GLU116
PTGS2	4RS0	6.77	-153.39	-109.64	-211.65	-23.78	4	SER451 THR212 ASN382 VAL447 HIS386 THR206
EGFR	3W33	6.26	-152.79	-65.87	-264.18	-25.15	3	ASP800 CYS797 MET793
PIK3CA	5SXI	5.44	-131.05	-53.18	-205.12	-19.61	5	SER673 GLU710 HIS676 ASP843 GLN475
FGF2	4OEE	4.70	-64.71	-25.24	-124.03	-2.58	2	LYS125 ARG120 GLY133 LYS135 ASN27
MAPK14	2ZAZ	4.39	-119.26	-70.21	-148.48	-14.61	5	GLY31 ASP168 GK1362 LYS53
CASP3	1GFW	3.91	-104.75	-31.03	-147.58	-19.57	3	ASP40 SER36 ASN35 ASP34
IL2	1M4A	2.44	-94.43	-0.90	-142.15	-10.22	4	LYS35 ARG38 LEU72

### Validation the Efficacy of Baicalin Against CS

**FIGURE 5:**
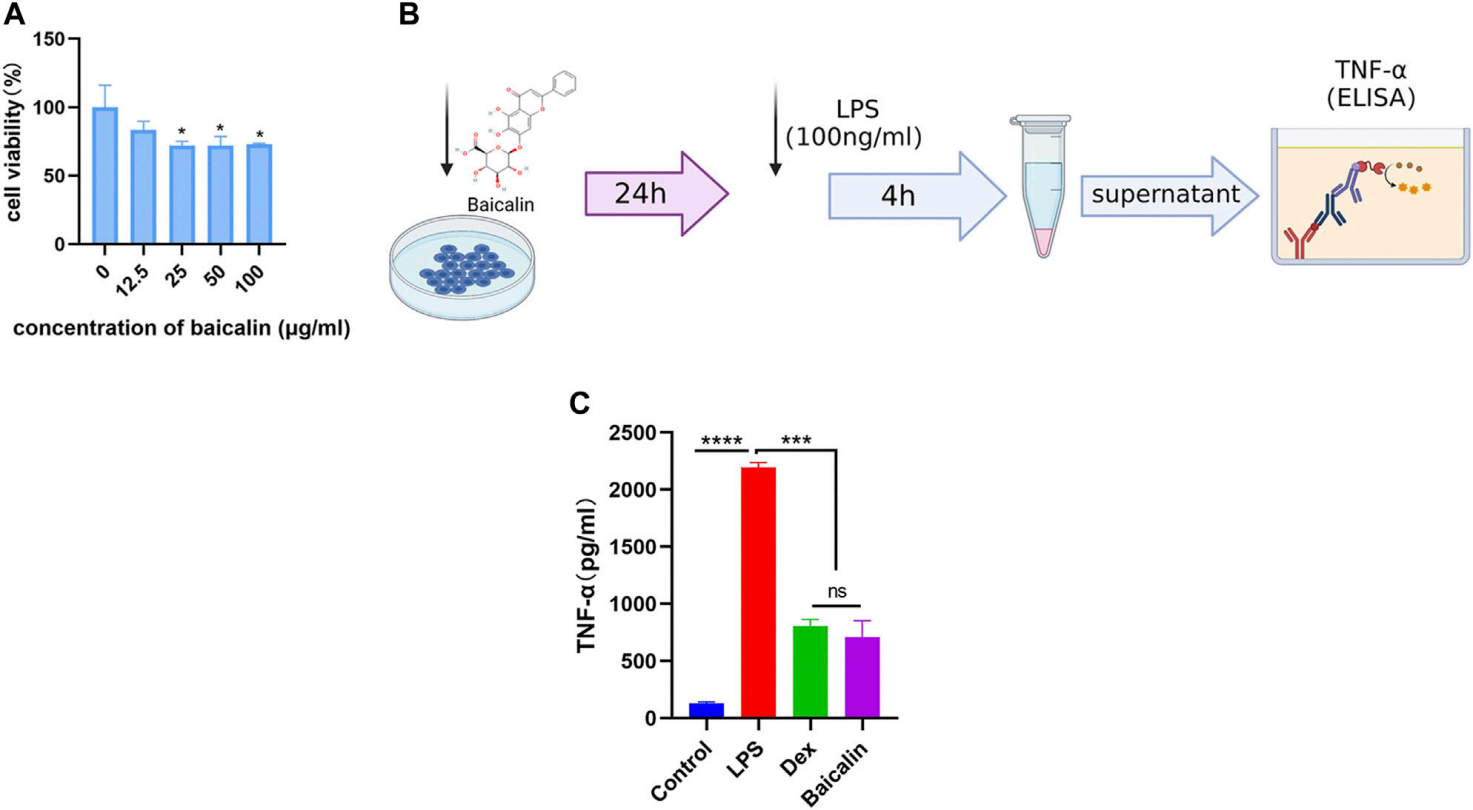
Baicalin inhibited LPS-induced TNF-α production *in vitro*. **(A)** Survival rate of MH-S cells after different concentrations of baicalin intervention for 24 h (Compared with 0 μg/ml baicalin, ^∗^ *p* < 0.05). **(B)** Scheme of LPS-induced macrophage activation. **(C)** Baicalin reduced the concentration of TNF-α released from LPS-activated macrophages.

**FIGURE 6:**
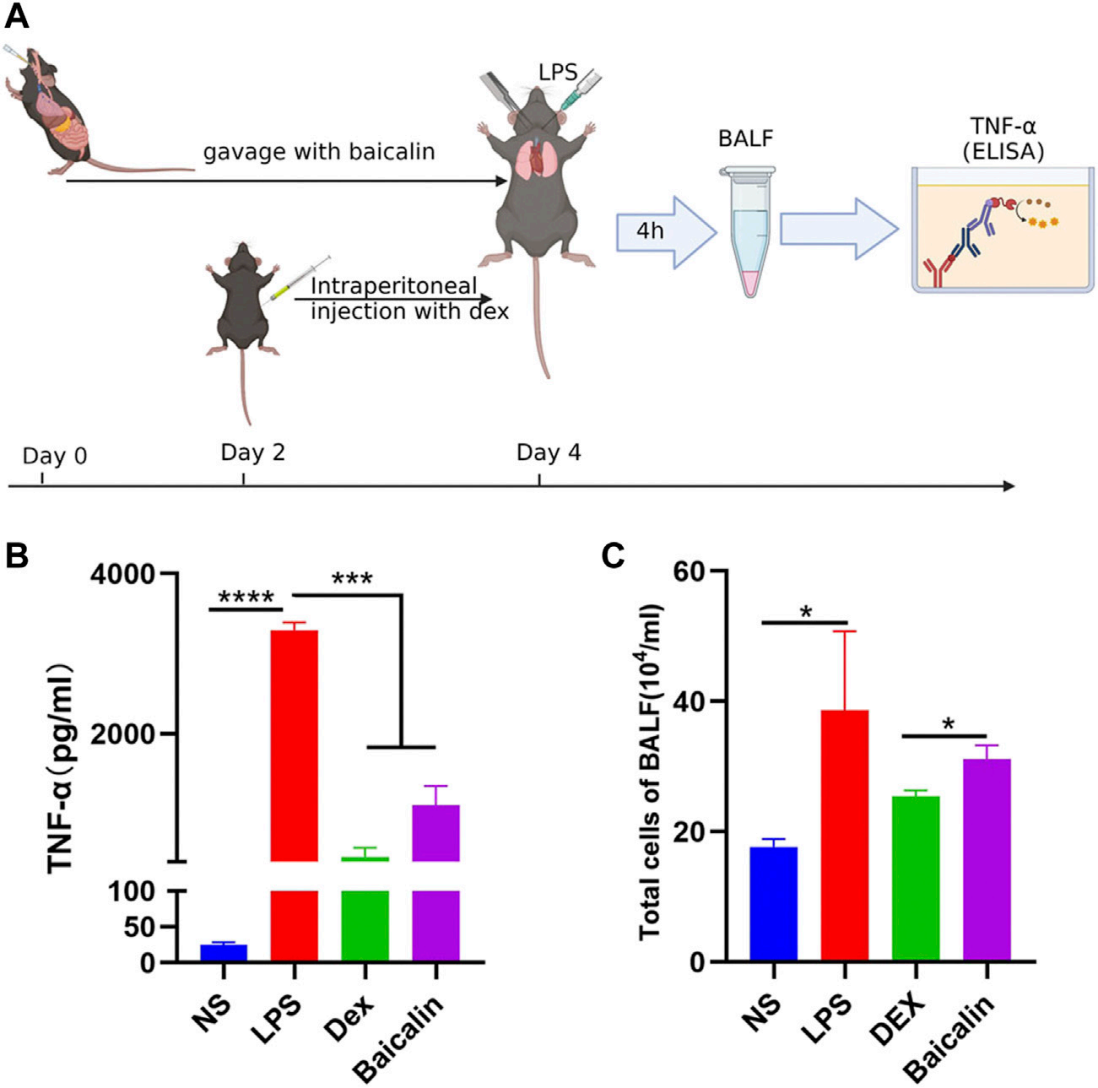
Baicalin inhibited LPS-induced TNF-α production *in vivo*. **(A)** Scheme of LPS-induced CS model *in vivo*. **(B)** Baicalin reduced the concentration of secreted TNF-α in LPS-induced CS. **(C)** Baicalin decreased the exudation of cells in BALF.

As shown in Figure 5A, the viability of MH-S cells gradually decreased with the increase of baicalin concentration, and 12.5 μg/ml baicalin had no significant effect on cell survival. In order to avoid the direct killing effect of baicalin on MH-S, we performed the CS model with 10 μg/ml (<12.5 μg/ml) *in vitro* (Figure 5B). It was demonstrated that baicalin significantly inhibited the secretion of TNF-α cytokines, as did Dex (Figure 5C). Furthermore, we also illustrated that baicalin significantly decreased the exudation of inflammatory cells and reduced the secretion of TNF-α in mouse BALF (Figure 6). Thus, TNF-α is indeed a potential target of baicalin action on CS as consistent with the results of the network analysis and deserves to be explored further.

### Potential Mechanisms of Baicalin’s Effects on CS and Key Signal Pathways

**FIGURE 7:**
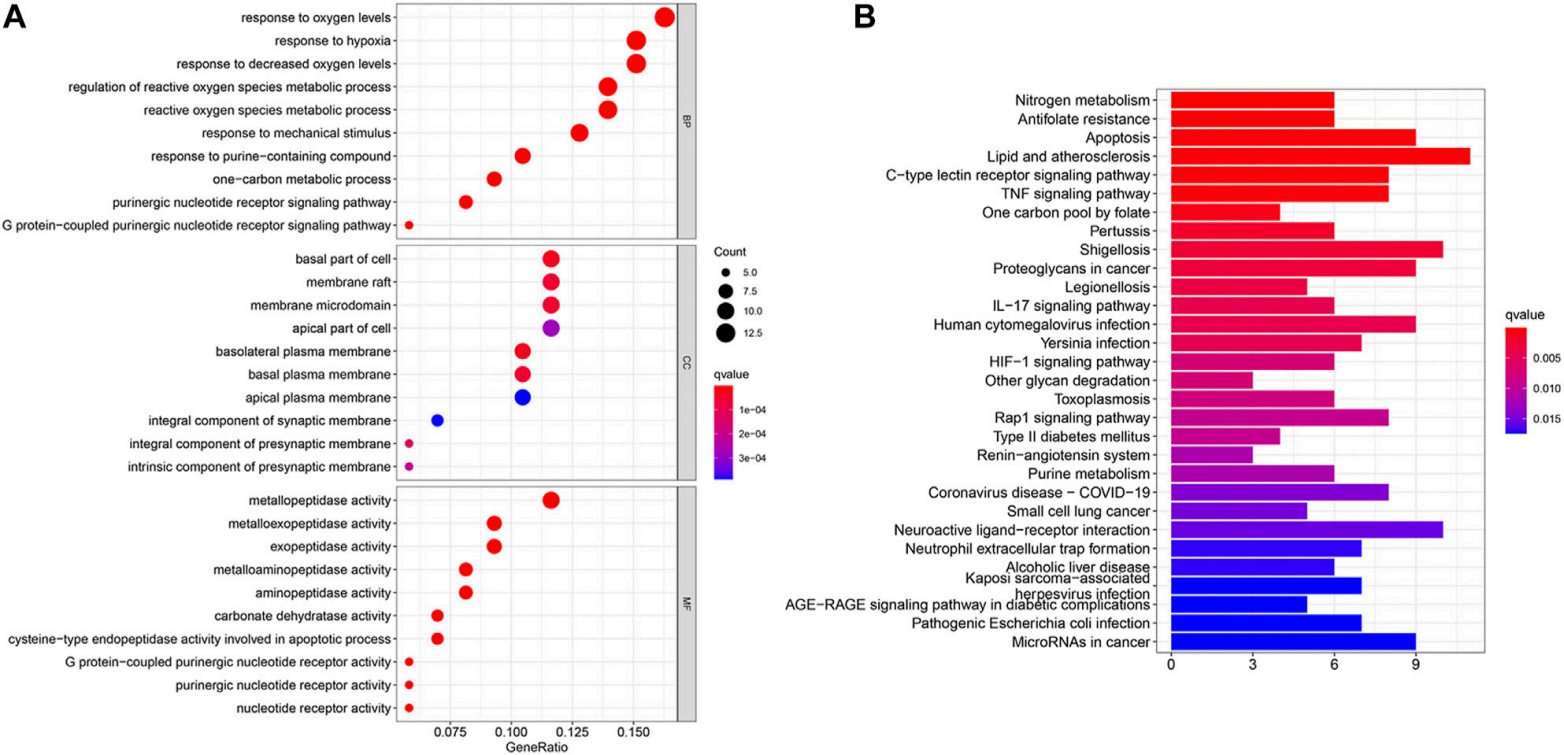
GO and KEGG enrichment analysis. **(A)** In the GO analysis bubble diagram, the vertical coordinates indicate the names of biological process (BP), cellular component (CC) and molecular function (MF), respectively, and the horizontal coordinates indicate the degree of enrichment. **(B)** In the KEGG functional enrichment graph, the vertical coordinate represents the pathway name and the horizontal coordinate represents the number of genes enriched by the pathway.

**FIGURE 8:**
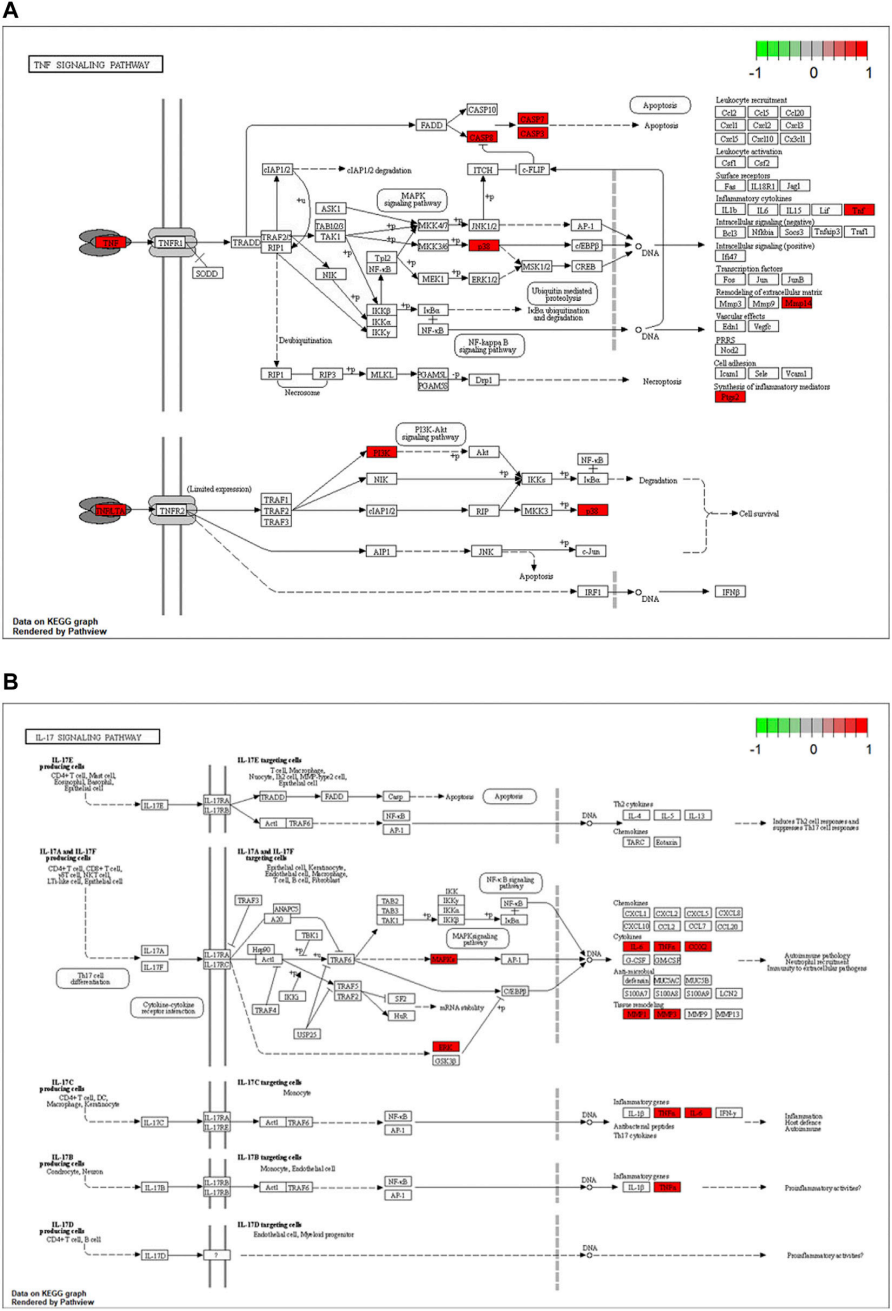
Critical KEGG pathways identified for baicalin action on CS. **(A)** Map04668: TNF signaling pathway. **(B)** Map04657: IL-17 signaling pathway. Arrows indicate upstream and downstream relationships between genes. Red represents the up-regulated common target gene in the network.

The potential biological functions of baicalin and CS mutual targets were identified by GO functional enrichment analysis, in which the top 10 markedly enriched BP, CC and MF classifications were presented in Figure 7A and Supplementary File S2. BP was mainly associated with response to oxygen levels, reactive oxygen species metabolic process, and response to hypoxia. Among the CC, the significantly altered pathways were basal part of cell, basolateral plasma membrane, basal plasma membrane, membrane raft, and membrane microdomain. Moreover, the MF of these genes were primarily performed in metallopeptidase activity, exopeptidase activity, and nucleotide receptor activity. Simultaneously, KEGG enrichment analysis identified the possible signaling pathways for the effect of baicalin on CS, mainly including antifolate resistance, TNF signaling pathway, IL-17 signaling pathway, Coronavirus disease-COVID-19, and Neutrophil extracellular trap formation, etc. (Figure 7B and Supplementary File S3). The location of overlapping genes in the key pathways were listed in Figure 8.

## Discussion

Baicalin is the main flavonoid component of the botanical *Scutellaria baicalensis Georgi*, native to several Asian countries (Zhao et al., 2016). It is well-known for its anti-inflammatory, anti-bacterial, anti-viral, antioxidant, and anti-tumor pharmacological effects (Dinda et al., 2017). Baicalin played an anti-inflammatory role by regulating the polarization of macrophages and the p38 MAPK signaling pathway to decrease the levels of pro-inflammatory cytokines IL-1, IL-6, IL-23, and TNF-α, and promote the expression of IL-10 and interferon regulatory factor 4 (IRF4) proteins (Yan et al., 2016; Zhu et al., 2016; Dinda et al., 2017). In addition, baicalin can also regulate the function of receptors associated with inflammation. Toll-like receptors (TLRs) are a family of ligand-binding receptors widely recognized in organisms that initiate the host immune response. Baicalin significantly reduced the levels of TNF-α, IL-1β, and IL-6 in depression-like hippocampal tissues by upregulating PI3K/AKT/FOXO1 pathway and inhibiting TLR4 expression and so alleviate neuroinflammation-induced depression-like behavior (Guo et al., 2019).

Baicalin has also been increasingly featured in the treatment of various viral-induced inflammatory diseases. A study indicated that baicalin could effectively inhibit the neuraminidase enzyme of influenza virus A/FM1/1/47 (H1N1), suppressing the virus replication, improving lung injury score, and prolonging mice life span (Ding et al., 2014). Moreover, baicalin could regulate the TLR7/MyD88/NF-κB axis to inhibit the expression of proinflammatory cytokines (TNF-α, IL-6, and IL-1β) which were induced by influenza virus A (H1N1) (Wan et al., 2014; Jiang et al., 2020). The decrease of IL-6 levels in supernatant via TLR4/NF-κB signaling pathway by baicalin was also observed in RSV-infected RAW264.7 cells (Yun CW et al., 2013). Furthermore, baicalin contributed to modulate the inflammatory response that caused by cytokines to be transient but not intense, preventing the worsening of immune-mediated damage (Pang et al., 2018).

With the exception of autoimmune diseases and bacterial infections, CS can be triggered by numerous viruses, including herpes viruses, such as herpes simplex virus, and influenza viruses, such as H1N1 and COVID-19 (Schulert et al., 2016; Fajgenbaum and June 2020). The ongoing prevalence of COVID-19 has affected more than 200 countries worldwide, even causing deaths. The latest variant of SARS-CoV-2, Omicron (B.1.1.529), has been classified by the World Health Organization (WHO) as a new coronavirus variant of concern (VOC) on 26 November 2021. It has been discovered to have higher transmissibility and greater pathogenicity, and significantly reduced effectiveness of the new coronavirus vaccine (He et al., 2021). Higher levels of granulocyte colony-stimulating factor (GCSF), interferon gamma-inducible protein 10kd (IP-10), monocyte chemotactic protein 1 (MCP-1), macrophage inflammatory protein 1A (MIP1A), TNF-α, IL-7, and IL-10 were also detected in the serum of patients with severe COVID-19 compared to patients with mild disease (Huang et al., 2020; Zhu et al., 2020; Yang et al., 2021). Furthermore, it was demonstrated that CS was not only strongly associated with the development of ARDS in COVID-19, but was also a crucial point in the progression of COVID-19 (Ye et al., 2020). In contrast, ongoing vaccine and antiviral drug development cannot catch up with the mutation rate of SARS-CoV-2. Therefore, preventing and treating CS would be a robust and effective strategy to reduce COVID-19 severity and mortality.

Encouragingly, a recent study identified good binding affinity of baicalin to the major targets of SARS-CoV-2, 3-trypsin-like cysteine protease (3CLpro) and papain-like protease (PLpro), using pharmacophore fitting and molecular docking techniques (Lin et al., 2021). It suggested that baicalin may inhibit proteases and reduce CS through inflammation-related pathways. However, it was not matched for CS-related targets or validated *in vivo*. Therefore, in order to further clarify the effect of baicalin on CS, we screened and obtained baicalin-related active targets and CS-related target genes from the Swiss Target Prediction database and other online databases. And, eighty-six genes were identified as co-target genes for baicalin and CS. Then, PPI network results showed that MAPK14, IL2, FGF2, CASP3, PTGS2, PIK3CA, EGFR, and TNF were the core gene targets of baicalin action on CS. MAPK14, a member of the p38 subfamily, was known to regulate inflammatory cytokines, and exerted an essential role in inflammatory acute lung injury (Pan et al., 2019). Fibroblast growth factor 2 (FGF-2) is a member of the family of transcriptional regulatory fibroblast growth factors. It was a regulator of apoptosis, angiogenesis, and involved in the inflammatory response (Laddha and Kulkarni, 2019; Cheng et al., 2020). The COVID-19 patients were detected with elevated FGF-2 in serum, which was closely associated with severe COVID-19 and ICU admission (Petrey et al., 2021). Caspase-3 in a family of cysteine-aspartate proteases was a terminal executor of endogenous and exogenous apoptosis (Yagami et al., 2019). Prostaglandin-endoperoxide synthase 2(PTGS2), the official gene symbol for Cyclooxygenase 2(COX-2), which can be rapidly and robustly expressed in a variety of pro-inflammatory cytokines and mediators, was a pivotal factor in the pathobiology of respiratory diseases such as inflammatory dysregulation (Rumzhum and Ammit, 2016). COX-2 is also a key mediator of inflammation, and the epidermal growth factor receptor (EGFR) could regulate its expression (Wang et al., 2017). In addition, EGFR dimerizes with HER-3 to catalyze the PI3K pathway, which played an important role in cell growth, and apoptosis resistance (Talukdar et al., 2020). It was associated with lung inflammation, and activated EGFR can lead to a pro-inflammatory response, including IL-8 expression in human airway epithelial cells (Wu et al., 2015). Besides, activation of PIK3CA, an essential kinase in the PI3K/AKT1/MTOR pathway, would evoke secretion of pro-inflammatory cytokines and migration of inflammatory cells into the lung (Ding et al., 2020). These genes were identified in our protein interaction network analysis. Thus, it is possible that baicalin suppress SARS-CoV-2-induced apoptosis to attenuate lung inflammation and effectively remedy cytokine storms via the above core gene targets. Interestingly, TNF was on the list of the most significant hub-genes for CS inhibition, as it had the highest number of interactions. Moreover, it was also the main interacting anti-inflammatory protein with baicalin in our studies. This coincided with TNF being considered as a therapeutic target for COVID-19 (Mehta et al., 2020).

TNF-α is a potent pro-inflammatory cytokine secreted by various immune cells and functions by binding to its receptors (TNFR) located throughout the body, with macrophages and monocytes being the prime sources of TNF-α that responds to inflammatory stimuli (Pennica et al., 1984; Aggarwal, 2003). It was reported that severe COVID-19 cases had high levels of pro-inflammatory cytokines in the germinal center, which could have generated a high-quality antibody response to establish long-term immunity, including TNF-α (Karki et al., 2021). TNF-α performed a pivotal role in the CS the same way as IL-6 and might be a major contributor to the increased severity of the disease. Moreover, TNF interaction with TNFR also led to increased expression of other cytokines (IL-6 and IL-1) and chemokines (Menegatti et al., 2019; Kovalchuk et al., 2021). Thereby, regulation of TNF-α production would be a crucial pathway for the prevention and therapy of CS. Inspiringly, molecular docking of the eight core genes with baicalin revealed that TNF-α had the highest docking score and strongest binding to baicalin. It indicated that baicalin had the potential to regulate TNF-α, a critical cytokine in the CS, which was also confirmed in both subsequent cellular, and animal experiments. Baicalin promisingly emerges as a representative drug for reducing the occurrence of CS and decreasing severe COVID-19.

LPS, a kind of bacterial endotoxin, is commonly applied to construct classical inflammation research models including sepsis, acute lung injury, and ARDS. It could provoke CS by activating monocytes and macrophages to produce high levels of pro-inflammatory cytokines (TNF-α, IL-6, and IL-1β, etc.), causing a systemic inflammatory response and even deaths (Zhao et al., 2019; He et al., 2020). So, LPS is also being widely utilized in the modeling of COVID-19-associated CS (Molinaro et al., 2020; Su et al., 2020). In addition, TNF-α was found at higher levels in the blood and diseased tissues of patients with severe COVID-19 and it was the critical cytokine in the occurrence of CS (Giamarellos-Bourboulis et al., 2020; Gong et al., 2020; Huang et al., 2020; Qin et al., 2020). In this study, we established a COVID-19-related CS model with LPS *in vitro* and *in vivo*, and the results demonstrated that baicalin could effectively inhibit the elevation of TNF-α, attenuate pulmonary inflammatory infiltration and suppress CS, which was consistent with the results of the above network analysis and molecular docking.

Furthermore, we explored the possible mechanisms of baicalin action on CS by GO and KEGG enrichment analysis. It revealed that numerous co-target genes were tightly correlated with inflammation-related signaling pathways, including TNF signaling pathway, IL-17 signaling pathway, and the currently known Coronavirus disease-COVID-19 infection pathway. The TNF signaling pathway has been demonstrated beneficially for the treatment of autoimmune and other chronic inflammatory diseases (Dorhoi and Kaufmann, 2014). It acted as an essential player in the systemic manifestations of inflammation, participating in the initiation, progression and termination of various stages of inflammation (Juhász et al., 2013). And, TNF is also a critical cytokine that can trigger a variety of intracellular signaling pathways, including inflammatory cell death, apoptosis, and immunity (Chu, 2013; Karki et al., 2021). Furthermore, TNF induced the production of IL-6 and other cytokines, involved in the process of CS in COVID-19 (Schett et al., 2020). As indicated by the KEGG signaling pathway map, TNF signaling induced the activation of many genes and mediated the inflammatory response through the NF-κB pathway, MAPK cascade and JNK pathway. IL-17 signaling performed pivotal roles in the CS caused by ARDS of the various causes and was associated with both alveolar inflammation and poor prognosis, as well as being a marker in the severity of viral infection (Mikacenic et al., 2016; Mahallawi et al., 2018). In addition, IL-17 was a potent inflammatory cytokine inducer which stimulated NF-κB and invoked NF-κB-dependent cytokines to exert pro-inflammatory effects (Onishi and Gaffen, 2010; Amatya et al., 2017). Besides, COVID-19 was defined as a highly infectious respiratory infection caused by SARS-CoV-2. Its main mechanism of occurrence was that SARS-CoV-2 infected alveolar epithelial cells via ACE2 receptors, activating the immune system and NF-kappa B pathway, which resulted in coronavirus disease 2019 (COVID-19), followed by the occurrence of CS (Battagello et al., 2020; Perico et al., 2021). Taken together, these results revealed that baicalin may intervene in the treatment of COVID-19-related CS through multiple immune target signaling pathways, in which NF-κB may be the main key target downstream of each signaling pathway. It is also to provide directions for the next studies.

Overall, with the combination of network analysis and molecular docking techniques, this study illustrated the hub targets and possible mechanisms of baicalin in treating CS, and also validated our prediction results to some degree by *in vitro* and *in vivo* experiments. Unfortunately, the specific mechanism of action of baicalin remains incomplete, and we have to conduct further experiments to further explore it. This study can provide the theoretical basis and research direction for the treatment of cytokine storm syndromes induced by a broad number of infections, including pandemic diseases like COVID-19.

## Conclusion

TCM encompasses a great number of drugs with potential in combating the SARS-CoV-2 pandemic by inhibiting CS, and reducing the proportion of serious disease COVID-19 patients. Besides, TCM has been recommended by the National Health Commission of China as a therapeutic drug in the guidelines for diagnosis and treatment of COVID-19 (the seventh edition, 03/04/2020), such as Lian Hua Qing Wen Capsules (National Health C, 2020). In our study, we also presented positive features of multi-pathway and multi-target effects of baicalin to inhibit CS, and initially unveiled the mechanism of baicalin in inhibiting CS through TNF signaling pathway. Furthermore, it might contribute to the subsequent screening of other herbal compounds with clear efficacy and provide a therapeutic direction for the global COVID-19 epidemic, including even pandemics like COVID-19 in the future. It also might deliver insight into the fundamental role of TCM in CS prevention and treatment.

## Data Availability

The original contributions presented in the study are included in the article/Supplementary Material, further inquiries can be directed to the corresponding authors.

## Supplementary Material

Supplementary File S1 The detailed information on the 86 overlapping target genes of baicalin and CS.

Supplementary File S2 Top ten overlapping gene ontology (GO) terms between baicalin targets and CS enriched in BP, CC, and MP categories.

Supplementary File S3 The detailed KEGG pathway information of baicalin on CS.
